# Nutrition, Anabolism, and the Wound Healing Process: An Overview

**Published:** 2009-02-03

**Authors:** Robert H. Demling

**Affiliations:** ^a^Harvard Medical School, Burn and Trauma Center, Brigham and Women's Hospital, Boston, MA

## Abstract

**Objective:** To develop a clear, concise, and up-to-date treatise on the role of anabolism from nutrition in wound healing. Special emphasis was to be placed on the effect of the stress response to wounding and its effect. **Methods:** A compilation of both the most important and most recent reports in the literature was used to also develop the review. The review was divided into sections to emphasize specific nutrition concepts of importance. **Results:** General and specific concepts were developed from this material. Topics included body composition and lean body mass, principles of macronutritional utilization, the stress response to wounding, nutritional assessment, nutritional support, and use of anabolic agents. **Conclusions:** We found that nutrition is a critical component in all the wound healing processes. The stress response to injury and any preexistent protein-energy malnutrition will alter this response, impeding healing and leading to potential severe morbidity. A decrease in lean body mass is of particular concern as this component is responsible for all protein synthesis necessary for healing. Nutritional assessment and support needs to be well orchestrated and precise. The use of anabolic agents can significantly increase overall lean mass synthesis and directly or indirectly improves healing by increasing protein synthesis.

Optimum nutrition is well recognized to be a key factor in maintaining all phases of wound healing. There are 2 processes that can complicate healing. One is activation of the stress response to injury, and the second is the development of any protein-energy malnutrition (PEM).

Any significant wound leads to a hypermetabolic and catabolic state, and nutritional needs are significantly increased. The healing wound depends on adequate nutrient flow (Fig 1). Of particular concern is the presence of any PEM, PEM being defined as a deficiency of energy and protein intake to meet bodily demands. PEM in the presence of a wound leads to the loss of lean body mass (LBM) or protein stores, which will in and of itself impede the healing process. Early aggressive nutrient and micronutritional feeding is essential to control and prevent this process from developing. PEM is commonly seen in the chronic wound population, especially the elderly, disabled, or chronically ill populations where chronic wounds tend to develop.^1^–^5^

Hunter, in 1954, followed by Culbertson and Moore, identified the fact that a wound being a threat to human existence takes preference for the available nutrients to heal, especially amino acids, at the expense of the host LBM.^6^–^8^ This process leads to an autocannibalism of available LBM to obtain the necessary amino acids for the required protein synthesis in the wound. If inadequate intake is present to keep up with needs, then PEM can develop. If inadequate glucose is available for the healing wound, proteins will break down into amino acids and through the alanine shunt lead to glucose synthesis by the liver. However, with severe losses of LBM, the host takes preference over the wound.^9^–^13^

This entire process is the result of the activation of the “stress response” to injury or wounding with its hormonal imbalance favoring body protein catabolism for substrate, needed for protein synthesis. There is also increased metabolic or calorie demand.^9^–^13^

There is a fundamental difference between the adequate intake seen in the unstressed patient and one where trauma or infection has activated the host stress response.^14^,^15^ Starvation alone produces a self-protective hormonal environment, which spares LBM with more than 90% of calories obtained from fat.^14^–^16^

To optimize healing, a substrate that is more dependent on intake than on the bodily breakdown of protein needs to be available. Chronic wounds are more complicated because the biology of the healing process is significantly altered. However, a stress response is activated with any wound and any existing PEM will accentuate the already poor healing process.^17^–^19^ For the above reasons, one cannot dissociate the normal process of healing from the nutritional status.

## BODY COMPOSITION AND LBM

### Components of body composition

To better understand the impact or erosion of LBM and the normal or abnormal utilization of protein and fat for fuel, a general understanding of normal body composition is required^19^–^21^ (Table 1).

Body composition can be divided into a fat and a fat-free component or LBM. LBM contains all of the body's protein content and water content, making up 75% of the normal body weight. Every protein molecule has a role in maintaining body homeostasis. Loss of any body protein is deleterious. The majority of the protein in the LBM is in the skeletal muscle mass. LBM is 50% to 60% muscle mass by weight.

It is the loss of body protein, not fat loss, that produces the complications caused by involuntary weight loss. Protein makes up the critical cell structure in muscle, viscera, red cells, and connective tissue. Enzymes that direct metabolism and antibodies that maintain immune functions are also proteins. Skin is composed primarily of the protein collagen. Protein synthesis is essential for any tissue repair. Therefore, LBM is highly metabolically active and necessary for survival.

**Figure 2:**
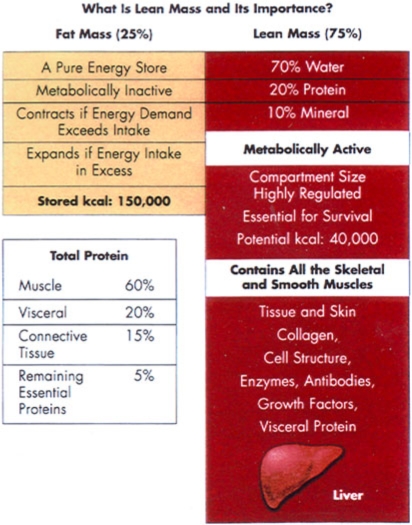
Body composition is divided into lean mass containing all the protein in the body plus water and fat mass composed mainly of a fat store, for a deposition of excess energy.

There are only 40,000 calories in the LBM compartment in a 70-kg individual; each gram of protein generates 4 calories (Fig 2). It is not possible to burn more than 50% of LBM.^22^ Fat mass comprises about 25% of body composition. For all intents, the fat compartment is a calorie reservoir where day-to-day excess calories are stored and fat is removed when demands need to be met. There are, however, some necessary essential fats, which make up a small fraction of this compartment.

For the most part, fat is not responsible for any essential metabolic activity. This energy reservoir contains about 110,000 calories stored, as 1 g of fat generates 10 calories (Fig 2). There are a number of body adaptations that attempt to maintain normal LBM or body protein (Table 2).^23^

There is an ongoing homeostatic drive to preserve LBM as a self-protective process since lost protein is deleterious. However, activation of the stress response, caused by a wound, will block these adaptive responses and body protein will be burned for fuel.^6^–^9^

### Measuring body composition (common approaches)

Involuntary weight loss is a marker of potential problems, and weight restoration is a potential solution. However, the real key diagnostic information is the status of body composition (Table 3). Since normal body composition for the individual of concern is not known prior to the insult, a host of normalized tables and equations, with an assumed normal value, are used. Therefore, the actual alteration of body composition caused by an insult or poor nutrition (or usually both) is not known. The complications, for example, the weakness seen in the patient, as well as the presence of a catabolic state that will lead to LBM loss, are often the best clinical markers. Of the available methods (Table 3), skin-fold thickness and bioelective impendence are valuable if taken sequentially over time, but some form of baseline is needed; on the other hand, nitrogen balance provides direct information as to whether the patient was catabolic or anabolic on the measurement day, and how catabolic.^22^–^28^

### Loss of LBM

Loss of any LBM is deleterious as there are no spare proteins. The loss of LBM, relative to normal, corresponds with major complications. A loss of more than 15% of total will impair wound healing, the greater the loss, the more the healing deficit. A loss of 30% or more leads to the development of spontaneous wounds such as pressure ulcers, and wound dehiscence at a late stage. Death occurs with 40% LBM loss, usually from pneumonia (Table 4).^22^

**Figure 3:**
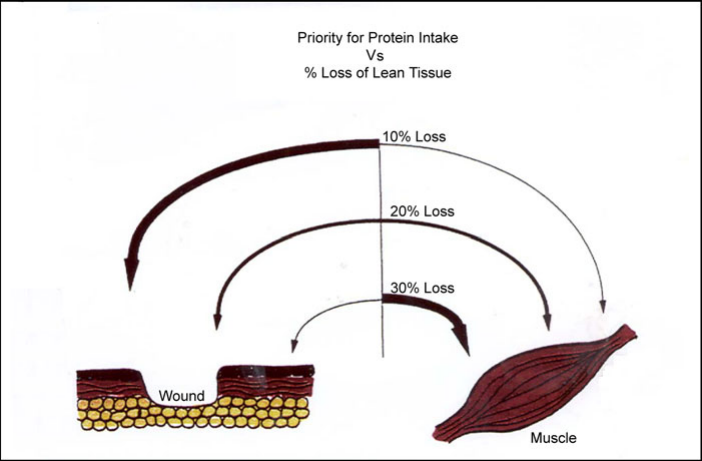
With a loss of lean mass less than 10%, the wound takes priority over the available protein substrate. As lean mass decreases, more consumed protein is used to restore LBM, with less being available to the wound. Wound healing rate decreases until lean mass is restored. With a loss of lean mass exceeding 30% of total, spontaneous wounds can develop due to the thinning of skin from lost collagen.

This table assumes no preexisting involuntary weight loss.^17^,^18^ Someone with PEM will always have a preexisting loss, which needs to be added as part of total. One can assume that with any stress-induced PEM, LBM loss is about half of the involuntary weight loss. The relationship between LBM and wound healing is based on the manner of utilization of available protein for either the wound or maintaining the overall LBM compartment (Fig 3).

Wound closure is an important genetically determined drive for survival. With a loss of less than 20% of LBM, the wound takes priority for the protein for healing. With a loss of 20%, there is equal competition for the protein between the wound and the restoration of LBM, so the healing rate will slow down. With a loss of 30% or more, where risk to survival is high, the LBM takes complete priority for protein intake. The wound essentially stops healing till LBM is restored at least partially.^6^–^8^

**Figure 4:**
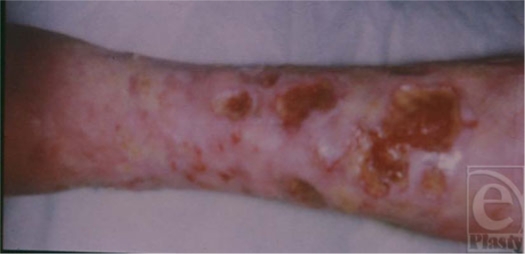
Lean mass loss 20% of the total: Clean but poorly healing acute wound responding to LBM loss.

**Figure 5:**
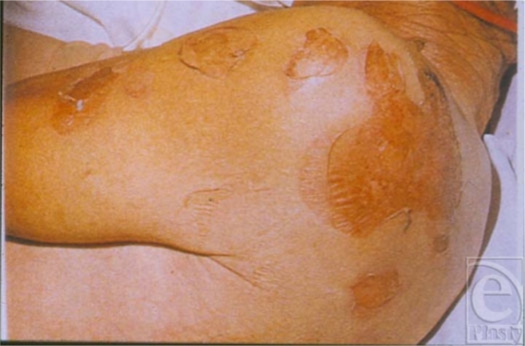
Lean mass loss 25% the total: thinning of skin with loss of collagen as LBM decreases.

**Figure 6:**
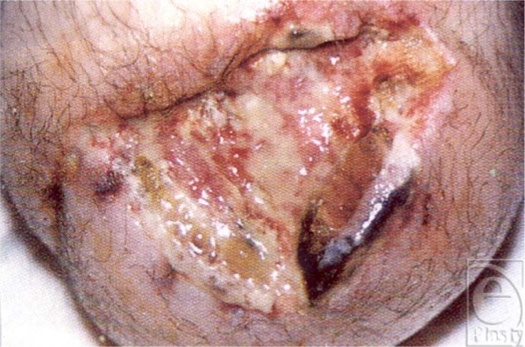
Lean mass loss 25% to 30% of the total: dehiscence stump closure now with open nonhealing wound.

**Figure 7:**
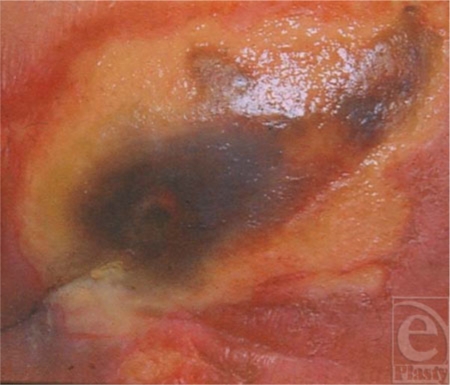
Lean mass loss 30% of the total: spontaneous pressure ulcer on the sacrum.

Body compositional changes before and after a wound, therefore, have a major impact on healing irrespective of the local wound care. In addition, nutritional support needs to be increased in both calories and protein (1.5 g/kg body weight) if there is a preexisting deficit, as would be present with any previous PEM. The rate of healing is directly related to the rate of restoration of body composition (Fig 3). Wound healing is directly related to the degree of LBM loss (Figs 4–7).^29^

## PRINCIPLES OF MACRONUTRIENT UTILIZATION (ADAPTIVE METABOLISM

Before discussing the principles of nutritional support for healing, it is important to understand the normal utilization of nutrients and the normal metabolic pathways to energy production and protein synthesis, which maintain the LBM compartment.^30^–^37^

Understanding the metabolic concept of macronutrient nutrient partitioning into an energy and protein compartment and methods to optimize an efficient nutrient channeling into either energy production or protein synthesis is the first step to understanding the nutritional support principles. In addition, the role of anabolic agents becomes clearer when considering their role as agents channeling protein substrate in protein synthesis.

In general, normal metabolism is directed by hormones that adjust when needed to and alter energy production to meet needs and also to restore daily protein balance through the natural tissue synthesis and breakdown pathways.^30^,^31^,^34^–^37^

### Energy pathway

Normally, the energy pathway is fueled almost completely by carbohydrates and fat.^31^–^33^

### Protein pathway

**Figure 8:**
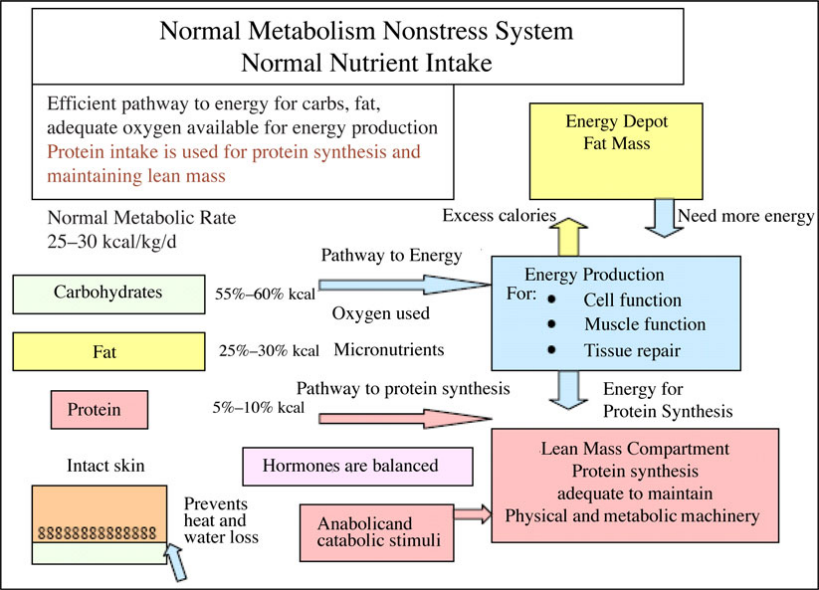
Macronutrients enter the metabolic pathways directly by hormones. Carbohydrates and fats enter the energy system or are stored as fat, while more than 90% of consumed protein enters the protein synthesis process. Normal skin prevents any energy drain through a wound.

Protein when consumed is metabolized into amino acids and peptides. With normal anabolic hormone activity, nearly all of the protein by-products are used for protein synthesis, not for energy. Only 5% is typically used for energy. However, energy is required for the protein synthesis process (Fig 8).^34^–^37^

**Figure 9:**
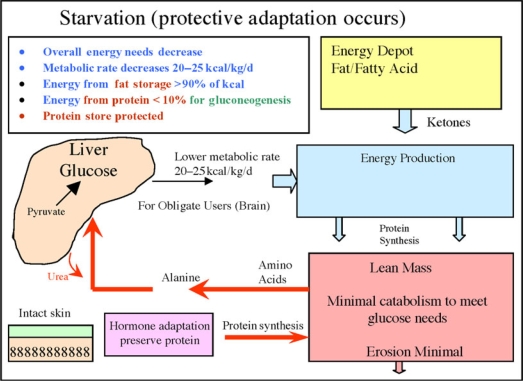
Starvation mode: protection of LBM. Hormone adaptation increases fat use for fuel with energy demands being decreased overall. A minimal amount of gluconeogenesis occurs to only maintain glucose to obligate users. LBM is in large part preserved.

With starvation, there is preservation of LBM compartment, as the majority of the calories come from the fat mass and only about 5% from protein.^16^,^33^ Metabolic rate and energy demands are decreased, cortisol levels (catabolic) decrease, and human growth hormone (HGH) levels (anabolic) increase (Fig 9).

## THE “STRESS RESPONSE” TO WOUNDING

The host response to severe illness or infection is an amplification of the fright-flight reaction.^11^,^12^,^38^–^40^ The insult leads to the release of inflammatory mediators that activate a very abnormal (Table 5) hormonal response, led by a marked increase in catecholamines and other hormones that produce a hypermetabolic-catabolic state.^38^–^41^

**Figure 10:**
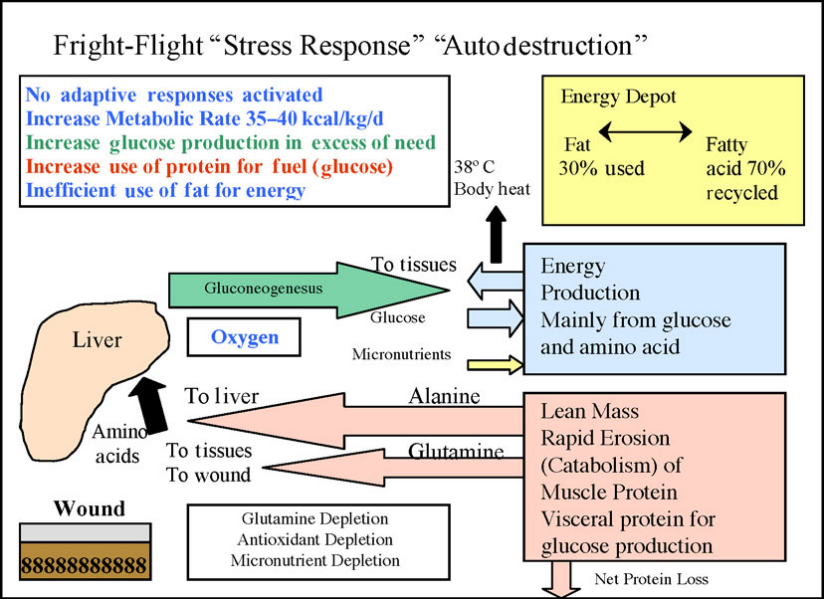
There is an overall increase in energy demands. Glucose production by the liver is markedly increased because of a hormonally driven process by amino acids from reabsorbed lean body mass. There is a net catabolic state. Energy demands are not selectively obtained from the fat deposit. Excess energy production is converted into excess body heat released through the skin. There is no protection of lean mass during this process.

An entire spectrum of abnormalities can be seen after injury and inflammation due to degrees of the manifestation of the host “stress response” to a body wound. If uncontrolled, the stress response can progress with loss of body protein and impaired wound healing. The once protective response then becomes autodestructive, and intense autocannibalism (catabolism for fuel) occurs with rapid loss of LBM^38^–^41^ (Fig 10).

Controlling the degree of ongoing injury requires both controlling the host response and at the same time supporting the metabolic needs to avoid further deterioration. However, catabolism still outweighs anabolism as the catabolic hormones predominate and the anabolic hormones, growth hormone, and testosterone are still decreased. Massive protein depletion can occur in days to weeks after a severe injury with wounds until the wound has been closed and the stress response has been removed.^14^,^38^–^44^

## NUTRITIONAL ASSESSMENT

The maintenance of optimum nutrition in the presence of a wound or PEM is a multifactorial process. Assessment has the following objectives (Tables 5–8).^31^,^45^

## ASSESSING THE NUTRITIONAL NEEDS

To optimize substrate flow to the healing wound, an assessment of required intake is made. There are many present values, which have been scientifically defined over the past 3 decades (Table 7).

There are a number of specific processes that need to be completed before the calories and protein intake can be determined. Assessment of nutritional needs can be divided into the following 3 components^46^–^48^ (Tables 6 and 8):

• Energy or calorie requirements

• Protein requirements

• Micronutrient requirements

### Calculation of energy needs

Daily energy expenditures (calories used) can be calculated or directly measured.^49^–^52^ Calculation is usually the preferred approach for the outpatient as the requirement for direct measurement is often available only in an acute care setting. Direct measurement using the method of indirect calorimetry is the most precise approach.^51^,^52^

The first step in calculating energy expenditure is to determine the basal metabolic rate (BMR) using predictive equations.^49^–^52^ This value reflects the energy to maintain homeostasis at rest shortly after awakening and in a fasting state for 12 to 18 hours^49^–^54^ (Table 6).

Usually, the basal or resting energy expenditure is about 25 kcal/kg ideal body weight for the young adult and about 20 kcal/kg for the elderly. Requirements for the injured or ill patient are usually 30% to 50% higher.^49^–^54^

*Malnourished patients*, who already have a deficit and have lost weight, require a 50% increase over calculated maintenance calories (energy).^47^,^55^–^57^

The second step is to adjust the BMR for the added energy caused by the “stress” from injury and wounds.^47^,^52^–^57^ This value, expressed as a present increase over the BMR, is an estimate of the value found for a number of bodily insults. The metabolic rate (energy demands) increases 20% after elective surgery and 100% after a severe burn.^47^,^48^,^52^–^57^ A wound, an infection, or a traumatic injury will fall between these 2 extremes. One simple formula for defining the stress factor is described below (Table 9). The stress factor is the multiplier of the BMR.^44^,^45^,^48^ The relative increase in the BMR has been defined for a number of disease processes. The data have been converted into a stress factor increase in the BMR (Table 9).

The third step is to determine the physical activity level of the patient. Physical activity is added by multiplying by an activity factor: for patients out of bed, 1.2 and for active exercise, 1.5 or more. Thus, the energy requirements can be calculated as follows:

\[
\hbox{Energy expenditure} = \hbox{BMR} \times \hbox{stress factor} \times \hbox{activity factor}^48
\]

**Table:** 

Malnourished patients, who already have a deficit and have lost weight require a 50% increase over calculated maintenance calories (energy).

### Indirect calorimetry

The reference standard for measuring energy expenditure in the clinical setting is indirect calorimetry. Indirect calorimetry is a technique that measures oxygen consumption and carbon dioxide production to calculate resting energy expenditure since 99% of oxygen is used for energy production. Oxygen used can be converted into calories required.^51^,^52^

### Protein requirements

After determining caloric (energy) requirements, protein requirements are assessed. A healthy adult requires about 0.8 g of protein per kilogram of body weight per day or about 60 to 70 g of protein to maintain homeostasis, that is, tissue synthesis equals tissue breakdown. Stressed patients need more protein, in the range of 1.5 g of protein per kilogram of body weight per day.^47^,^48^,^58^–^63^ The increased needs stem from both increased demands for protein synthesis and increased losses of amino acids from the abnormal protein synthesis channeling where protein substrate is also used for fuel. Urinary nitrogen losses increase after injury and illness, with an increase in the degree of stress. Nitrogen content is used as a marker for protein (6.25 g of protein is equal to 1 g of nitrogen). Nitrogen balance studies, such as a 24-hour urinary urea nitrogen measurement, that compare nitrogen intake with nitrogen excretion can be helpful in determining needs by at least matching losses with intake. Nutritionally depleted but nonstressed patients, especially the elderly, also require 1.5 g/kg/day to restore the lost body protein.^59^–^63^ Stressed, depleted patients usually cannot metabolize more than 1.5 g/kg/day of protein unless an anabolic agent is added, which can override the catabolic stimulus. The required protein intake for a number of clinical states has been defined and can be used as estimates (Table 10). Simply, aging increases protein requirements to avoid sarcopenia.

### Micronutrient support

Micronutrients are compounds found in small quantities in all tissues. They are essential for cellular function and, therefore, for survival. It is becoming increasingly clear that marked deficiencies in key micronutrients occur during the severe stress response or with any superimposed PEM as a result of increased losses, increased consumption during metabolism, and inadequate replacement.^64^–^68^ Because micronutrients are essential for cellular function, a deficiency further amplifies stress, metabolic derangements, and ongoing catabolism.

The micronutrients include organic compounds (vitamins) and inorganic compounds (trace minerals). These compounds are both utilized and excreted at a more rapid rate after injury, leading to well-documented deficiencies. However, because measurement of levels is difficult, if not impossible, prevention of a deficiency is accomplished only by providing increased intake. Deficiency states can lead to severe morbidity. Specific properties of these important molecules will be described later. Although the doses of the various micronutrients required to manage wound stress are not well defined, a dose of 5 to 10 times the recommended daily allowance is recommended until wound stress is resolved and the wound has healed.^47^,^64^–^68^ There are specific micronutrients required for wound healing. Replacement in sufficient amounts is essential (Table 11).

## NUTRITIONAL SUPPORT: THE PROCESS

### Macronutrient distribution

Once the assessment is complete and the nutrient needs in terms of calories and protein intake are made, macro- and micronutrients are provided. Macronutrients include carbohydrate, fat and protein. In the presence of a large traumatic wound or a burn, the stress response has been activated requiring an increase in calories for energy and protein for protein synthesis. The breakdown for feeding a catabolic state is described as follows.

Approximately 55% to 60% of total calories should be delivered as complex carbohydrates instead of simple sugars. Each gram of carbohydrate generates 3.3 kcal. Excess carbohydrates will lead to hyperglycemia, a major complication resulting in impeded healing and immune dysfunction. Maximum glucose utilization is considered to be 7 μg/kg/min.^48^,^57^

Approximately 20% to 25% of calories should be provided by fat, but not more than 2 g/kg/day. Values in excess will likely not be cleared from serum. Triglyceride levels should be kept below 250 mg/dL. Fat provides 10 kcal/g.

Because normal protein preservation in LBM is not maintained with a wound stress response, approximately 20% to 25% of total calories need to be provided as protein. Inadequate intake will not prevent protein use for calories, as LBM becomes the source.

### Carbohydrates and wound healing

As described, calories are needed to supply the energy needed to heal and carbohydrates are the key source of energy through lactate use. Skin cells are dependent on glucose for energy. In patients with diabetes, careful control of glucose intake, with adequate insulin, is essential to optimizing healing rate.

Carbohydrates have also been shown to be important for a wound unrelated to energy production. These carbohydrate factors include structural lubricant, transport, immunologic, hormonal, and enzymatic functions. Carbohydrates are a key component of glucoproteins, which is a key element in the healing wound used for its structure and communicative properties. Carbohydrates have also been found to be a key factor in the activity of the enzymes hexokinase and citrate synthase used for wound-repair reactions.^69^–^72^

Cell adhesion, migration, and proliferation is regulated by cell-surface carbohydrates including B-4-glycosylated carbohydrate chains.^72^ Glucose is also used for inflammatory cell activity leading to the removal of bacteria and of necrotic material (Table 12).^73^

Lactate is a metabolic byproduct of glucose. This 2-carbon compound appears to have many important wound healing effects. The increase in wound lactate is required for the release of macrophage angiogenesis factor. Lactate stimulates collagen synthesis by fibroblasts and is an important activator of the genetic expression of many healing pathways in addition to its role as an energy source.^74^,^75^

### Fats and wound healing

Fats are unique in that they function both as a source of energy and also as signaling molecules. It is important to recognize that the composition of cell membrane basically reflects dietary fatty acid consumption. Cell membrane composition affects cell-function-influencing enzyme absorption such as protein kinase C and a variety of genes. White adipose tissue is a source of proinflammatory fat metabolism and is one of the key regulators of wound inflammation and healing.^76^–^84^

Fats are broken down into free fatty acids and then packaged into chylomicron absorption and transportation to the body for energy or storage. The essential fatty acids must be consumed in the diet. Polyunsaturated fatty acids are used for cell membrane production while saturated fatty acids are often used for fuel.^77^–^80^ The oxidative stress typical in the inflamed wound can lead to membrane alteration by a process called lipid peroxidation, which can alter wound cell function. In addition, circulating by-products can have a negative affect by stimulating wound cell death or apoptosis, while other lipid by-products such as leptins protect the cell.^77^–^80^

It is clear, however, that adequate fat, whether consumed or obtained from the fat depot by lipase activity, is essential to wound healing of both acute and chronic wounds. The first role is to provide adequate energy to the wound. The second role is to provide the substrate for the many roles of fat by-products, especially the components of free fatty acids on wound cell function, wound inflammation, and wound cell proliferation. At present, it would appear that a dietary intake containing high levels of monosaturated fatty acids and omega-3 polysaturated fatty acids is ideal. Lipid components are responsible for tissue growth and wound remodeling including collagen and extracellular matrix production.^84^–^91^

It can be seen that fat and its derived lipid products are an extremely diverse class of molecules, which includes fatty acids and all their metabolic derivatives. Fats are a major source of energy in addition to its role as various signaling molecules.^80^–^91^

### Protein and wound healing

It is well recognized that protein is required for wound healing and a protein deficiency retards healing in both acute and chronic wounds.^57^,^59^,^63^,^87^–^97^ This fact is particularly evident in chronic pressure ulcers and acute burn injury. Dipeptides and polypeptides have been shown to have a wound healing activity. Several amino acids, such as leucine, glutamine, and arginine, all have anabolic activity. It has been shown that there is a greater protein accretion with orally fed protein, which becomes a hydrolysate than parenteral protein, which consists of total breakdown into amino acids.

The renewal of the skin involves 2 components: cell proliferation, mostly fibroblasts and protein synthesis, mainly collagen from the fibroblasts. Both components require protein substrates.^89^,^93^ After injury, both metabolic processes are accelerated to repair the wound. In a severely injured patient, with a wound, the metabolic process for healing must occur in the presence of a hypermetabolic catabolic state.^47^,^48^,^57^,^59^–^62^ This state will cause a protein malnutrition very rapidly if a high protein intake is not rapidly initiated. However, an injured man can use only a certain amount of protein. Also, severely burned adults can assimilate only 1.5 g/kg/day into the LBM, additional protein will only be used as a fuel source, unless anabolic activity is increased.

It has been found that in a major injury, skin is in a negative protein status identical to the net whole-body loss of protein.^90^–^92^ Use of an anabolic stimulus like insulin and provision of an adequate amino acid supply can control this deleterious process.^89^–^93^ Modulation of anabolic factors will not only improve the whole-body protein balance but will also increase the skin protein metabolism.^89^–^93^ Positive skin-protein synthesis will accelerate the wound healing process.

### Glutamine

Glutamine is the most abundant amino acid in the body and accounts for 60% of the intracellular amino acid pool.^98^ This amino acid is considered to be conditionally essential as a deficiency can occur rapidly after injury. Glutamine is used as an energy source after the stress response as it is released from cells to undergo glucose conversion in the liver for use as energy.^98^,^99^ In addition, glutamine is the primary fuel source for rapidly dividing cells like epithelial cells during healing.

Glutamine has potent antioxidant activity, being a component of the intracellular glutathione system. It also has direct immunological function by stimulating lymphocyte proliferation through its use as energy. Glutamine has anticatabolic and anabolic properties also and is the rate-limiting agent for new protein synthesis (Table 13).

Because of its many roles in the wound, it is of particular concern when there is a rapid fall in both intracellular and extracellular glutamine levels, to a deficiency state, in the presence of a major wound. Replacement using a glutamine dose of 0.3 to 0.4 g/kg/day is commonly performed after a major burn.^100^,^101^ Of interest is that glutamine delivery at this level has been shown to increase survival after major burns.^100^,^101^ Glutamine supplementation in and of itself has not been shown to dramatically impact the wound. However, it does appear to decrease wound infection and it does improve healing in experimental studies.^102^–^104^ Glutamine intake of 2 g or more does increase HGH release, which has potent anabolic activity. In general, it is clear that glutamine does assist in restoration and maintenance of LBM and that property in and of itself will improve healing.

Excess glutamine provision is deleterious. Since this amino acid has 2 nitrogens and is metabolized into ammonia, excess will increase the risk of increased ammonia levels and azotemia. This process is more prominent in the elderly population where added glutamine exceeds the metabolic pathways for glutamine use and excess is, therefore, metabolized.

### Zinc

Zinc is a cofactor for RNA and DNA polymerase and is, therefore, involved with DNA synthesis, protein synthesis, and cell proliferation. Zinc is a key cofactor for matrix metalloproteinase activity and is also involved in immune function and collagen synthesis. Zinc is also a cofactor for superoxide dismutase, an antioxidant (Table 14). After wounding, there is a redistribution of body zinc with wound levels increasing and levels in normal skin decreasing.

The hypermetabolic state leads to a marked increase in urinary loss of zinc, and a risk for a zinc deficiency state has adverse effects on the healing process including a decrease in epithelial rate, wound strength, and decreased collagen strength. Restoration of the expected zinc deficiency state is usually performed by oral provision of zinc sulfate 220 mg tid.^105^,^106^–^109^

There are data that would indicate that correction of a zinc deficiency is beneficial while zinc supplementation over and above replacement has no added benefit in wound healing. However, zinc supplementation is a common approach to managing wounds.

### Arginine

Arginine is another conditionally essential amino acid whose level decreases after major trauma and wounds.^97^,^110^–^112^ Arginine has been shown to stimulate immune function and is used for a variety of components of healing including a proline precursor. Its role in wound healing itself has not been clearly defined, although large doses have been shown to increase tissue collagen content. High doses also stimulate the release of HGH. It has recently been shown that the healing effect is not due to nitric oxide synthesis.^97^,^104^,^111^–^113^

### Other micronutrient support

Micronutrients are required for cofactors in energy production and protein synthesis. Since energy demands are increased, cofactor needs are also increased.^105^–^109^,^114^–^138^ The various micronutrients and their roles and estimated requirements are presented for the presence of a large wound. The key vitamins for energy are the B complex and vitamin C, water-soluble vitamins that need to be replaced daily^105^,^114^–^122^ (Table 15).

The micronutrients involved in energy production are described. Vitamin B complex is a prominent factor. Zinc is very prominent as it is a cofactor for a large number of enzymes involved in DNA synthesis and is protein synthesis.^105^,^107^,^114^

The micronutrients required for anabolic and anticatabolic activity and protein synthesis are described in Table 16*. These elements have properties considered to be directly involved with protein synthesis and as cell protectors through potent antioxidant properties. Oxidants are a major source of cell toxicity with wound inflammation, and antioxidant activity is essential for the wound healing process to continue. Vitamin C and glutathione, products of glutamine, are water-soluble antioxidants. (Table 17) Other vitamins and minerals with antioxidants activity are described in Table 16.

There are now well-recognized micronutrients that are necessary for anabolic activity and that can actually improve net protein synthesis (Table 17). These components include the amino acids glutamine and arginine already described. A variety of vitamins and microminerals are also involved in this process.

Increased anabolic and wound healing benefits have also been shown for the conditionally essential amino acids, glutamine, and arginine. Both of these amino acids characteristically decrease with activation of the stress response leading to a deficiency state well recognized to impede protein synthesis and overall anabolism.*. Replacement therapy has been shown in both circumstances to increase net anabolism.

The trace elements that have clear healing properties include zinc, copper, and selenium. Copper is a key factor for overall homeostasis. It is necessary for a cofactor for antioxidant activity to control oxidant stress, assisting in energy formation in the respiratory chain at cytochrome c. In addition, copper is used for collagen and elastin cross-linking. By 10 days after severe injury serum, copper levels are decreased. It is probably an increase in the acute-phase protein ceruloplasmin that leaks into the tissues taking copper with it. Copper replacement therapy is often performed after major wounds like burns. Typically, 1 to 2 mg of copper is provided.^105^,^114^

Manganese is associated with various enzymes in the Krebs Cycle and is also involved with protein metabolism. It also activates lipoprotein lipase and also protein synthesis. Manganese, Mn, is also a cofactor for the antioxidant superoxide dismutase and also for metalloproteinase activity in the wound. A deficiency state after severe trauma or in the presence of a large burn is yet to be documented. Maintenance dosing is 0.3 to 0.5 mg daily.

Selenium is required for the glutathione system to work, glutathione being the major intracellular antioxidant. Management of the wound-inflammation-induced oxidant stress is a key component of cell protection during the healing process. Selenium is excreted in increased amounts in the urine after major injury.^108^

Muscle contains almost half the total body selenium. Myositis coupled with myocardiopathy is seen clinically with selenium deficiency. Replacement is common after burns and severe trauma including wounds, at a daily dose of 100 to 150 mg.

## ANABOLIC HORMONE ADJUNCTIVE THERAPY TO NUTRITION

As described, there are a number of key hormones involved with energy production, catabolism, and anabolism, all directly or indirectly affecting wound healing. The stress response to injury leads to a maladaptive hormone response, producing an increase in the catabolic hormones and a decrease in anabolic hormones, growth hormones, and testosterone. The altered stress hormonal environment can lead to both a significant increase in catabolism, or tissue breakdown, and a decrease in the overall anabolic activity.^9^,^10^

It is now well recognized that the hormonal environment, so critical to wound healing, can be beneficially modified.^108^,^109^,^130^–^138^ In general, restoration or improvement in net protein synthesis and, therefore, in wound healing, is the result of 2 hormonal processes. The first is an attenuation of the catabolic hormonal response, and the second is an increase in overall anabolic activity, recognizing that adequate nutrition is being provided. Any hormonal manipulation that decreases the rate of catabolism would appear to be beneficial for wound healing. Blocking the cortisol response would seem to be intuitively beneficial and, as stated, growth hormone and testosterone analogues decrease the catabolic response to cortisol.

A number of clinical studies have demonstrated the ability of exogenous delivery of anabolic hormones to increase net nitrogen retention and overall protein synthesis. Wound healing has also been reported to be improved.* However, it remains unclear as to how much of the wound healing is the result of an overall systemic anabolic effect, or whether there is a direct effect on wound healing. Anabolic hormones for which data are available are listed in Table 18.

In subsequent sections, individual anabolic hormones will be discussed, including HGH, insulin-like growth factor (IGF), insulin, testosterone, and testosterone analogues, also known as anabolic steroids.

## HUMAN GROWTH HORMONE

HGH is a potent endogenous anabolic hormone produced by the pituitary gland. HGH levels are at there highest during the growth spurt, decreasing with increasing age. Starvation and intense exercise are 2 potent stimuli, while acute or chronic injury or illness suppresses HGH release, especially in the elderly. The amino acids glutamine and arginine, when given in large doses, have been shown to increase HGH release.

HGH has a number of metabolic effects (Table 19). The most prominent is its anabolic effect. HGH increases the influx and decreases the efflux of amino acids into the cell. Cell proliferation is accentuated, as are overall protein synthesis and new tissue growth. HGH also stimulates IGF-1 production by the liver, and some of the anabolism seen with HGH is that produced by IGF-1, another anabolic agent.^134^–^138^,^142^

The effect on increasing fat metabolism is beneficial in that fat is preferentially used for energy production, and amino acids are preserved for use in protein synthesis. Recent data indicate that insulin provides some of the anabolic effect of HGH therapy. At present, the issue as to the specific anabolic effects attributed to HGH versus that of IGF-1 and insulin remains unresolved.

Clinical studies have in large part focused on the systemic anabolic and anticatabolic actions of HGH. Populations in which HGH has been shown to be beneficial include severe burn and trauma. Increases in LBM, muscle strength, and immune function have been documented in its clinical use. Increase of anabolic activity requires implementation of a high-protein, high-energy diet.^136^–^138^,^142^–^144^

Significant complications can occur with the use of HGH. The anti-insulin effects are problematic in that glucose is less efficiently used for fuel and increased plasma glucose levels are known to be deleterious.

In summary, use of HGH in conjunction with adequate nutrition and protein intake clearly results in increased anabolic activity and will positively impact wound healing by increasing protein synthesis in catabolic populations.

### Insulin-like growth factor-1

IGF-1 is a large polypeptide that has hormone-like properties. The IGF-1, also known as somatomedin-C, has metabolic and anabolic properties similar to insulin. Practically speaking, this agent is not as much used for its clinical wound healing effect or anabolic activity as HGH or IGF. The main source is the liver, where IGF synthesis is initiated by HGH. Decreased levels are noted with a major body insult.^144^,^146^

Metabolic properties include increased protein synthesis, a decrease in blood glucose, and an attenuation of stress-induced hypermetabolism, the latter 2 properties being quite different from HGH. The attenuation of stress-induced hypermetabolism is a favorable property of IGF-1. The major complication is hypoglycemia.

### Insulin

The hormone insulin is known to have anabolic activities in addition to its effect on glucose and fat metabolism. In a catabolic state, exogenous insulin administration has been shown to decrease proteolysis in addition to increasing protein synthesis.^137^,^138^,^142^–^144^ The anabolic activity appears to mainly affect the muscle and skin protein in the LBM compartment. An increase in circulating amino acids produced by wound amino acid intake increases the anabolic and anticatabolic effect in both normal adults and populations in a catabolic state.

A number of clinical trials,^137^,^138^,^142^–^144^ mainly in burn patients, have demonstrated the stimulation of protein synthesis, decreased protein degradation, and a net nitrogen uptake, especially in skeletal muscle. The positive insulin effect on protein synthesis decreases with aging. There are much less data on the actions of insulin on wound healing over and above its systemic anabolic effect. The main complication is hypoglycemia.

### Testosterone analogues

Testosterone is a necessary androgen for maintaining LBM and wound healing. A deficiency leads to catabolism and impaired healing. The use of large doses exogenously has increased net protein synthesis, but a direct effect on wound healing has not yet been demonstrated. In general, it has relatively weak anabolic and wound healing properties.

Anabolic steroids refer to the class of drugs produced by modification of testosterone.^143^–^154^ These drugs were developed to take clinical advantage of the anabolic effects of testosterone while decreasing androgenic side effects of the naturally occurring molecule. The mechanisms of action of testosterone analogues are through activation of the androgenic receptors found in highest concentration in myocytes and skin fibroblasts. Some populations of epithelial cells also contain these receptors. Stimulation leads to a decrease in efflux of amino acids and an increase in influx into the cell. A decrease in fat mass is also seen because of the preferential use of fat for fuel. There are no metabolic effects on glucose production.

All anabolic steroids increase overall protein synthesis and new-tissue formation, as evidenced by an increase in skin thickness and muscle formation. All these agents also have anticatabolic activity decreasing the protein degradation caused by cortisol and other catabolic stimuli. In addition, all anabolic steroids have some androgenic or masculinizing effects.

The anabolic steroid oxandrolone happens to have the greatest anabolic and least androgenic side effects in the class of anabolic steroids.^147^–^149^ Most of the recent studies on anabolic steroids and LBM have used the anabolic steroid oxandrolone. Oxandrolone has potent anabolic activity, up to 13 times that of methyltestosterone. In addition, its androgenic effect is considerably less than testosterone, minimizing this complication common to other testosterone derivatives. The increased anabolic activity and decreased androgenic (masculinizing) activity markedly increase its clinical value. Oxandrolone is given orally, with 99% bioavailability. It is protein bound on plasma with a biologic life of 9 hours^149^ (Table 20).

The anabolic steroids, especially oxandrolone, have been successfully used in the trauma and burn patient population to both decrease LBM loss in the acute phase of injury as well as more rapidly restore the lost LBM in the recovery phase. Demonstrated in several studies is an increase in the healing of chronic wounds. However, significant LBM gains were also present.^153^,^154^

It is important to point out that in all of the clinical trials where LBM gains were reported, a high-protein diet was used. In most studies, a protein intake of 1.2 to 1.5 g/kg/day was used. The effects of anabolic steroids on wound healing appear to be, in a large part, due to a general stimulation of overall anabolic activity. However, there is increasing evidence of a direct stimulation of all phases of wound healing by these agents.^153^,^154^

The mechanism of improved wound healing with the use of anabolic steroids is not yet defined. Stimulation of androgenic receptors on wound fibroblasts may well lead to a local release of growth factors.

## CONCLUSION

Nutritional status is extremely important in wound healing, especially the major wounds. A common nutritional deficiency state is PEM, either that produced by the “stress” response to wounding or a preexisting state.^155^–^169^

Maintenance of anabolism and controlling catabolism is critical to optimizing the healing process. Increased protein intake is required to keep up with catabolic losses and allow wound healing anabolic activity. Micronutrients, carbohydrates, and fat are used predominately as fuel, but each has direct wound effects essential for healing. Protein as a micronutrient is inappropriately used for fuel after injury, so intake needs to be increased to allow for protein synthesis. There are also specific actions of protein by-products impeding the healing process.

Micronutrients are often ignored, but, as described, there are many essential metabolic pathways depending on the vitamins and minerals. Select amino acids such as glutamine are also essential. Of importance is the fact that increased losses of many micronutrients occur in the presence of a wound. In addition, increased daily requirements are needed to keep up with an increase in demands during the postinjury hypermetabolic state. Also, supplementation of compounds such as glutamine has not only been shown to improve wound and immune states but also to decrease trauma- and burn-induced mortality.

Finally, controlling catabolism by producing anabolism by agents, many being endogenous, has been shown in the presence of adequate protein intake to increase net body anabolism, which, in turn, will improve overall protein synthesis including the wound.

Anabolic hormones are necessary to maintain the increased protein synthesis required for maintaining LBM, including wound healing, in conjunction with the presence of adequate protein intake. However, endogenous levels of these hormones are decreased in acute and chronic illness and with increasing age, especially in the presence of a large wound. Because the lost LBM caused by the stress response, aging, and malnutrition retards wound healing, the ideal use of these agents is to more effectively restore anabolic activity. There are also data that indicate a direct wound healing stimulating effect for some of these hormones.

Recognition of all these principles will optimize the wound healing effects of nutritional support.

*References 94–103, 110–112, 114, 118,123–127, 139–141.

*References 97–103, 110–112, 139–141

*References 108, 109, 130–138, 142–154.

## Figures and Tables

**Figure 1:**
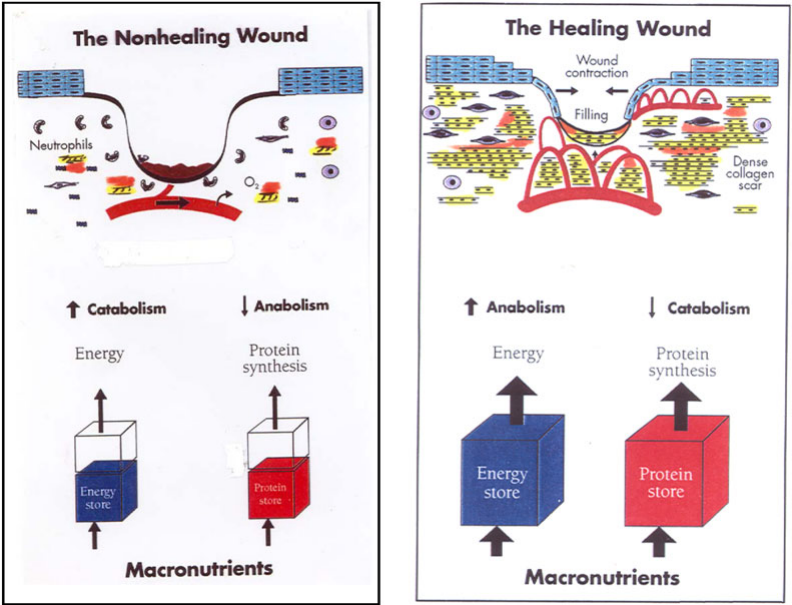
Balance between adequacy of macronutrients and net anabolism and catabolism and its impact on wound healing.

**Table 1:** Conditions associated with development of protein-energy malnutrition

Catabolic illness, “the stress response,” eg, trauma, surgery, wounds, infection, corticosteroids
Involuntary weight loss exceeding 10% of ideal, for any reason
Chronic illnesses, eg, diabetes, cancer, mental impairment, arthritis, renal failure
Wounds, especially chronic
Increase in nutritional losses; open wounds, enteral fistulas
Intestinal-tract diseases impairing absorption

**Table 2:** What maintains lean mass

Intense genetic drive to maintain essential protein stores
Anabolic hormones that stimulate protein synthesis
Resistance exercise
Adequate protein intake to meet the demands

**Table 3:** Methods routinely available to assess body composition

Method	Description	Advantage	Disadvantage	Precision (coefficient of variation), %
Measurement of skin-fold thickness	Thickness of subcutaneous	Easily performed with portable equipment	Possibility of error and interobserver variability in measurement	5–10
Bioimpedance analysis	Low-level current is introduced, and measurements of impedance are used to calculate fat and fat-free mass	Easily performed with portable equipment, used to calculate body cell mass	Results will be affected by hydration	<5
Nitrogen balance	Measurement of nitrogen intake minus urinary nitrogen loss to determine net nitrogen gain or loss	Easy to perform, indicates a real time evaluation of lean body mass	Not totally reliable, as there are other nitrogen losses besides urine	10–15

**Table 4:** Complications relative to loss of lean body mass*

Lean body mass (% loss of total)*	Complications (related to lost lean mass)	Associated, mortality, %
10	Impaired immunity, increased infection	10
20	Decreased healing, weakness, infection, thinning of skin	30
30	Too weak to sit, pressure sores develop pneumonia, no healing	50
40	Death, usually from pneumonia	100

*Assuming no preexisting loss.

**Table 5:** Major metabolic abnormalities with response to injury “stress response”

Increased catabolic hormones (cortisol and catechols)
Decreased anabolic hormones (human growth hormone and testosterone)
Marked increase in metabolic rate
Sustained increase in body temperature
Marked increase in glucose demands and liver gluconeogenesis
Rapid skeletal muscle breakdown with amino acid use as an energy source (counter to normal nutrient channeling)
Lack of ketosis, indicating that fat is not the major calorie source
Unresponsiveness of catabolism to nutrient intake

**Table 6:** Weight versus basal metabolic requirement

Body weight, kg	50	55	60	65	70	75	80
Normal basal metabolic rate, kcal/d	1310	1410	1600	1600	1700	1780	1870

**Table 7:** Objectives of nutritional assessment

Control the catabolic state
Restore sufficient macronutrient intake to meet current energy and protein needs
Increase energy intake to about 50% above daily needs, restore adequate calories to respond to wounding or to begin the process of weight and lean mass gain
Increase protein intake to 2 times the recommended daily allowance (0.8 g/kg/d), ie, to 1.5 g/kg/d to allow for restoration of wound healing and any lost lean body mass
Increase anabolic stimulation to direct the substrate from protein intake into protein synthesis
Avoid replacement of lost lean mass with fat gain
Utilize exercise (mainly resistance exercise) to increase the bodies anabolic drive to maintain and more rapidly regain lean mass
Consider use of exogenous anabolic hormones to increase net protein synthesis

**Table 8:** Calculation of energy expenditure (calories)

Determine BMR
Determine activity level as a fractional increase from BMR
Estimate stress factor (caused by wound)
Energy = BMR × stress factor × activity factor

BMR indicates basal metabolic rate.

**Table 9:** Calculation of stress factors

Stress insult	Stress factor
Minor injury	1.2
Minor surgery	1.2
Clean wound	1.2
Bone fracture	
Infected wound	1.5
Major trauma	
Severe burn	

**Table 10:** Protein requirements

Condition	Daily needs, g/kg/d
Normal	0.8
Stress Response	1.5–2
Correct protein-energy malnutrition	1.5
Presence of wound	1.5
Restore lost weight	1.5
Elderly	1.2–1.5

**Table 11:** Essential micronutrients for wound healing

Vitamins	
Vitamin A	Stimulant for onset of wound healing process
	Stimulant of epithelialization and fibroblast deposition of collagen
Vitamin C	Necessary for collagen synthesis
Minerals	
Zinc	Cofactor for collagen and other wound protein synthesis
Copper	Cofacter for connective tissue production
	Collagen cross-linking
Manganese	Collagen and ground substance synthesis

**Table 12:** Carbohydrate role in the wound

Energy production
Lubricant matrix
Transport
Immunologic
Hormonal
Enzymatic

**Table 13:** Properties of glutamine

1. Anabolic, anticatabolic
2. Stimulates human growth hormone release
3. Acts as Antioxidant
4. Direct fuel for rapidly dividing cells
5. Immune stimulant
6. Shuttle for ammonia
7. Synthesis for purine and pyrimidines

**Table 14:** Zinc properties

Cofactor for many protein synthesis pathways and DNA synthesis collagen production
Stimulates reepithelialization
Cofactor matrix metalloproteinase activity
Cofactor superoxide dismutase and glutathione with antioxidant activity
Augments immune function

**Table 15:** Arginine effects

Precursor of proline in collagen
Precursor for nitric oxide
Increases hydroxy proline production
Stimulates release of wound anabolic hormones insulin, insulin like growth factors, and human growth factors
Local immune stimulant for lymphocytes
A conditionally essential amino acid

**Table 16:** Anticatabolic and anabolic micronutrient support

Amino acids	
Glutamine	Decreases net nitrogen loss
	Increases net muscle protein synthesis
	Nitrogen carrier
	Stimulates human growth hormone release
Arginine	Decreases net nitrogen loss
Antioxidants	
Vitamins A, C, E, B; carotene, Zn, Cu, Se	Decreases net oxidant-induced protein degradation
Protein synthesis cofactors	
Zn, Cu, Mg, vitamin B complex	Improve protein synthesis pathways

**Table 17:** Micronutrient support of the hypermetabolic state: energy production

Vitamin B complex		Daily dose
Thiamine	Oxidation reduction reactions	10–100 mg
Riboflavin	Oxidative phosphorylation for adenosine triphosphate production	10 mg
Niacin	Electron transfer reactions for energy production	150 mg
Vitamin B_6_	Transamination for glucose production and breakdown	10–15 mg
Folate	One carbon transfer reaction required for all macronutrient metabolism	0.4–1 mg
Vitamin B_12_	Coenzyme A reactions for all nutrient use	50 μg
Vitamin C	Carnitine production for fatty acid metabolism	500 mg to 2 g
Minerals		
Selenium	Cofactor for fat metabolism	100–150 μg
Copper	Cofactor for cytochrome oxidase for energy production	1–2 mg
Zinc	Cofactor for DNA, RNA, and polymerase for protein synthesis	4–10 μg
Amino acids		
Glutamine	Nitrogen shuttle for glucose amino acid breakdown, urea production, direct source of cell energy	10–20 g

**Table 18:** Anabolic hormone activity

Increased anabolism	Direct wound effect
Insulin	Yes	Unclear
Human growth hormone	Yes	Unclear
Insulin-like growth factor-1	Yes	Yes
Testosterone	Yes	No
Anabolic steroids	Yes	Yes

**Table 19:** Metabolic effects of human growth hormone

Increases cell uptake of amino acids
Increases nitrogen retention
Increases protein synthesis
Decreases cortisol receptor activity
Increases releases of Insulin-like growth factor-1
Increases insulin requirements
Increases fat oxidation for fuel, decreasing fat stores
Increases metabolic rate (10%–15%)
Produces insulin resistance, often leading to hyperglycemia

**Table 20:** Clinical effect of anabolic steroids

Attenuate the catabolic stimulus during the “stress response”
More rapid restoration of lost lean mass
Restore normal nutrient partitioning
Improved healing of chronic wounds with restoration of lost lean mass
